# Gelatinases Cleave Dentin Sialoprotein Intracellularly

**DOI:** 10.3389/fphys.2020.00686

**Published:** 2020-06-25

**Authors:** Xiaohui Gou, Yifan Xue, Huiwen Zheng, Guobin Yang, Shuo Chen, Zhi Chen, Guohua Yuan

**Affiliations:** ^1^The State Key Laboratory Breeding Base of Basic Science of Stomatology & Key Laboratory for Oral Biomedicine of Ministry of Education, School and Hospital of Stomatology, Wuhan University, Wuhan, China; ^2^Department of Developmental Dentistry, University of Texas Health Science Center, San Antonio, TX, United States

**Keywords:** dentin sialoprotein, gelatinases, FURIN, odontoblasts, dentinogenesis

## Abstract

Dentin sialoprotein (DSP), the NH2-terminal fragment of dentin sialophosphoprotein (DSPP), is essential for dentin formation and further processed into small fragments inside the odontoblasts. Gelatinases, including matrix metalloproteinases 9 (MMP9) and MMP2, were able to cleave DSP(P) in tooth structures. We hypothesized that gelatinases may also cleave DSP intracellularly in the odontoblasts. In this study, the co-expression and physical interaction between DSP and gelatinases were proved by double immunofluorescence and *in situ* proximity ligation assay (PLA). Intracellular enzymatic activity of gelatinases was verified by gelatin zymography and *in situ* zymography. To confirm whether DSP was cleaved by active gelatinases intracellularly, lysates of *wild-type (WT)* odontoblastic cells treated with a MMP2 inhibitor or a MMP9 inhibitor or a MMP general inhibitor and of *Mmp9^–/–^* odontoblastic cells were analyzed by western blotting. Compared with the *WT* odontoblastic cells without inhibitor treatment, all these groups exhibited significantly higher ratios of high molecular weight to low molecular weight band density. FURIN was verified to be co-localized and physically interacted with MMP9 by double immunofluorescence and *in situ* PLA. The ratio of proMMP9 to activated MMP9 inside the odontoblastic cells were increased when function of endogenous FURIN was inhibited. And overexpressed proMMP9 was intracellularly cleaved by FURIN in the HEK293E cells, which was completely blocked by the mutation of proMMP9 with R^96^TPR^99^ substituted by A^96^AAA^99^. Taken together, these results indicate that DSP is intracellularly processed by gelatinases, and FURIN is involved in the intracellular activation of proMMP9 through cleavage of its R^96^TPR^99^ motif.

## Introduction

Dentin is a major mineralized tissue of tooth and dentinogenesis is an important process in tooth development. Multiple non-collagenous proteins (NCPs) secreted by odontoblasts are involved in dentinogenesis and biomineralization (Linde, 1989; Butler and Ritchie, 1995; Butler, 1998; Goldberg et al., 2011). Among these NCPs, dentin sialophosphoprotein (DSPP), is regarded as a key factor which exerts influence over the odontoblasts differentiation, maturation, secretion, and mineralization of dentin matrix (Prasad et al., 2010; Jia et al., 2015; Chen et al., 2016).

Only trace amount of full-length DSPP was found in the extracts of pulp/odontoblasts and dentin. And the majority of DSPP is proteolytically cleaved into the NH2-terminal dentin sialoprotein (DSP) and the COOH-terminal dentin phosphoprotein (DPP) shortly after the protein is synthesized (MacDougall et al., 1997; Sun et al., 2010). DSP is essential for dentin formation and tooth development, evidenced by hereditary dentin defects resulting from heterogeneous mutations in the coding region of DSP (Begue-Kirn et al., 1998; Baba et al., 2004; Malmgren et al., 2004; Lee et al., 2008; Ozer et al., 2013; Wan et al., 2016; Li et al., 2017). However, an *in vitro* experiment showed that full-length DSP has limited effects on apatite formation and growth (Boskey et al., 2000). Our previous work reported that DSP, similar to DSPP, is processed into NH2- and COOH-terminal fragments inside the mouse odontoblastic cells. Meanwhile, the expression patterns of NH2- and COOH-terminal fragments of DSP in mouse dentin were opposite in the dentin matrix. The NH2-terminal fragments of DSP were enriched in the non-mineralized predentin matrix but weak in the mineralized dentin, while the COOH-terminal fragments were mainly distributed in the mineralized dentin (Yuan et al., 2012). These results suggested that the majority of full-length DSP may be processed into smaller fragments by some unknown enzymes in the cytoplasm of odontoblasts before being transported into dentin matrix (Yuan et al., 2012).

Gelatinases (MMP9 and MMP2) are a subfamily of matrix metalloproteinases (MMPs) and are known to participate in numerous physiological and pathological events associated with mineralized tissues (Vu et al., 1998; Mazzoni et al., 2007; Nyman et al., 2011). MMPs may have an impact on dentin formation and mineralization, because widened predentin and impaired mineralized dentin were observed in the embryonic mouse tooth germs cultured with Marimastat (a general MMP inhibitor), and these dentinogenesis defects were also apparent when the lowest concentration of CT1166 (a more selective inhibitor of gelatinases) was used, indicating that gelatinases are possibly involved in the dentin formation (Fanchon et al., 2004). MMP9 is highly expressed in the odontoblasts (Mazzoni et al., 2007; Feng et al., 2012; Yuan et al., 2017), and our previous study reported that *Mmp9* null mice displayed aberrant tooth development similar to *Dspp*−/− mice with delayed differentiation of odontoblasts, widened predentin, and irregular mineralization front. We have also proved that DSP is a novel substrate of MMP9 (Yuan et al., 2017). MMP2 is also expressed in the odontoblasts, and may play a crucial role in accelerating dentin matrix mineralization by proteolytic processing of DPP (Caron et al., 2001; Satoyoshi et al., 2001). During dentinogenesis, DSPP is processed by MMP2 in the dentin extracellular matrix, and several cleavage sites of DSPP are near the DSP COOH-terminal region (Yamakoshi et al., 2006). Previously, gelatinases were thought to be synthesized and secreted as inactive zymogens, which were activated in the extracellular matrix and participated in various physiological and pathological events by degrading extracellular matrix components (Gupta, 2012; Vandooren et al., 2013). But recently, several studies have demonstrated that MMPs including MMP9 and MMP2 can also cleave intracellular substrates, such as apoptotic regulators, molecular chaperones, cytoskeletal proteins, and others (Cauwe and Opdenakker, 2010; Gupta, 2012). We hypothesized that MMP9 and MMP2 may be involved in cleaving DSP into fragments intracellularly in the odontoblasts.

Gelatinases are synthesized as latent zymogens with prodomains at the NH2-terminus and are processed into activated enzymes by removing their prodomains (Gacko, 2001; Kang et al., 2002). It is still not clear which proteinases are involved in the activation of zymogens of gelatinases in the odontoblasts. FURIN, a member of proprotein convertase (PC) family, is expressed in various types of cells, including the odontoblasts (Akamatsu et al., 2000; Thomas, 2002). FURIN has been shown to proteolytically activate various proprotein substrates throughout the secretory pathway, such as MMP11, MT1-MMP, MT3-MMP (Pei and Weiss, 1995; Yana and Weiss, 2000; Kang et al., 2002). FURIN recognizes and cleaves the consensus sequence -Arg-X-Lys/Arg-Arg↓-(RX(K/R)R↓) (where Lys is lysine, Arg is arginine, X = 0, 2, 4, 6…. and ↓represents the cleavage site) in the substrates (Thomas, 2002). We analyzed and found a potential cleavage motif of FURIN (R^95^TPR^98^↓ in human; R^96^TPR^99^↓ in mouse) in the prodomain of latent MMP9. We hypothesized that FURIN may cleave latent MMP9 at the R^96^TPR^99^ site, leading to the activation of latent MMP9 in mouse odontoblasts.

This study aimed to determine whether the full-length DSP was cleaved by activated MMP9 and MMP2 in the cytoplasm of odontoblasts, and to test the role of FURIN in activating MMP9 intracellularly.

## Materials and Methods

### Antibodies and Reagents

A rabbit polyclonal anti-mouse DSP (M-300 # sc-33587) antibody was purchased from Santa Cruz Biotechnology Inc. (Dallas, TX, United States). Anti-MMP9 (AF909) and Anti-MMP2 (AF1488) antibodies were purchased from R&D SYSTEMS (United States). A monoclonal anti-FURIN (ab183495) and a polyclonal anti-MMP9 (ab38898) antibodies were purchased from Abcam Corp. (Cambridge, United Kingdom). A mouse monoclonal anti-MYC-tag (60003-2-Ig), an anti-beta Actin (60008-1-Ig) and a monoclonal anti-GAPDH (60004-1-Ig) antibodies were purchased from Proteintech (United States). MMP9 Inhibitor I (444278) was purchased from EMD Millipore Corp. (Billerica, MA, United States). MMP2 Inhibitor II (444286) was purchased from EMD Chemicals Inc. (San Diego, CA, United States). MMPs broad-spectrum inhibitor used in the study was obtained from Thermo Fisher Scientific. FURIN Inhibitor I (Decanoyl-RVKR-CMK) (34493) was purchased from Merck Millipore (Darmstadt, Germany). IFKine^TM^ Red Donkey Anti-Rabbit IgG (A24421) and IFKine^TM^ Green Donkey Anti-Goat IgG (A24231) were purchased from Abbkine Scientific Co. (California, CA, United States). EnzChek^TM^ Gelatinase/Collagenase Assay Kit (E12055) was purchased from Thermo Fisher Scientific.

### Construction of Plasmids

The plasmid *pCMV3-mMmp9-Flag* (MG50560-CF) was purchased from Sino Biological (Beijing, China). The plasmids of *Furin* (Myc-DDK-tagged) (MR210693) and *Mmp3* (Myc-DDK-tagged) (MR207673) were purchased from ORIGENE (Beijing, China). A mutant plasmid expressing the mutant proMMP9 with R^96^TPR^99^ substituted by A^96^AAA^99^ was constructed using the Mut Express II Fast Mutagenesis Kit V2 (C214-01, Vazyme, China). The mutagenic forward primer (5′ to 3′: *ATTgcagcagcagcaTGTGGTGTCCCAGACGTGG*) and reverse primer (5′ to 3′: *ACAtgctgctgctgcAATGGCCTTTAGT GTCTGGCTG*) (lowercased nucleotides indicate the altered codon) were designed by CE Design V1.04.

### Animal and Tissue Preparation

All experimental procedures involving the use of animals were reviewed and approved by the Animal Welfare Committee at the School of Stomatology, Wuhan University. The detailed information about *Mmp9^–/–^* mice used in this research was described previously (Yuan et al., 2017). For animal studies, mice of postnatal day (PN) 1, 2, 3, and 7 were sacrificed and mandible tissues were dissected. All specimens were fixed in a solution of 4% paraformaldehyde (PFA) overnight, demineralized by 10% ethylenediaminetetraacetic acid, and dehydrated in a graded ethanol series. Paraffin-embedded sections were cut and prepared.

### Cell Culture and Transfection

DPCs extracted from *wild-type (WT*) and *Mmp9^–/–^* neonatal mice and HEK293E cells were maintained in dulbecco’s modified eagle medium/high glucose (DMEM) (catalog no. SH30022.01, GE Healthcare Life Sciences, United States) containing 20% fetal bovine serum (Invitrogen) and 1% penicillin/streptomycin (catalog no. SV30010, GE Healthcare Life Sciences, United States) at 37°C. When cells reached 80–90% confluence, plasmids were transfected into HEK293E cells using Lipofectamine 2000 (Invitrogen, China) according to the manufacturer’s instructions. Then, transfected HEK293E cells were incubated at 37°C for 2 days and cell lysates were collected for western blotting assay. When the cells confluent up to 80%, culture condition of DPCs was replaced with the odontoblastic-differentiation induction media containing 10% fetal bovine serum, 1% penicillin/streptomycin, 10 mM sodium β-glycerophosphate, 50 μg/ml L-ascorbic acid, and 10 nM dexamethasone. After culturing for 7 days, these cells were confirmed as odontoblastic cells by detecting the expression of odontoblast maker genes, including *Dspp*, *dentin matrix protein 1 (Dmp1)*, and *Collagen I* as described in our previous study (Zheng et al., 2020). Then the odontoblastic cells were prepared for immunofluorescence, *in situ* Proximity Ligation Assay (PLA), immunoprecipitation assay, and gelatin zymography.

To test gelatinases’ effect on DSP expression pattern inside the odontoblasts, *WT* odontoblastic cells were cultured with serum-free medium for 8 h and then divided into four groups randomly. In group 1 (control group), the cells were treated with DMSO; In group 2, the cells were treated with a MMP9 inhibitor II; In group 3, the cells were treated with a MMP2 inhibitor I; In group 4, the cells were treated with a MMPs broad-spectrum inhibitor. After culturing for additional 12 h, cell lysates were obtained for DSP western blotting.

### Immunofluorescence and Immunohistochemistry

For double immunofluorescence, *WT* odontoblastic cells plated on coverslips were washed three times with ice-cold phosphate-buffered saline (PBS), fixed in cold 4% PFA, and permeabilized in 0.1% TritonX-100. *WT* mouse mandible tissue sections were processed through deparaffinization, rehydration, and antigen retrieval. Then, the cells and sections were blocked with normal donkey serum (catalog no. ANT051, AntGene) for 1 h at 37°C, and primary anti-DSP (rabbit; 1:50) and anti MMP9 (goat; 1:50) antibodies were incubated simultaneously overnight at 4°C. The cells and sections incubated with negative IgG were applied as negative control. After washes with PBS, the cells and sections were incubated for 1 h at 37°C with Alexa Fluor 488 green donkey anti goat IgG and Alexa Fluor 568 red donkey anti rabbit IgG (1:150). DAPI was used for nuclear staining. Images were obtained using Leica DM4000B microscope. For FURIN immunohistochemistry, *WT* mouse mandible tissue sections were blocked with normal donkey serum and then incubated with primary anti-FURIN antibody (rabbit, 1:200) overnight. Sections were counterstained with hematoxylin and mounted.

### *In situ* PLA

Interaction of DSP with either MMP9 or MMP2 was detected by the DuoLink PLA kit (Olink Biosciences, Uppsala, Sweden; PLA probe anti-rabbit minus for the detection of the rabbit DSP antibody, catalog no. DUO92005; PLA probe anti-goat plus for the detection of the goat anti MMP9 or MMP2 antibody, catalog no. DUO92003; Detection Kit, catalog no. DUO92008) in the odontoblastic cells and PN2 mouse mandibular molars. After being blocked, Duolink *in situ* PLA was performed according to the manufacturer’s instructions. Briefly, the odontoblastic cells (seeded on coverslips) and the tissue sections of PN2 mandibles were incubated with primary anti-DSP polyclonal antibody (rabbit, 1:50) together with anti-MMP9 antibody (goat, 1:50) or with anti-MMP2 antibody (goat, 1:50) overnight at 4°C. Corresponding secondary antibodies conjugated to Duolink PLA probes (MINUS and PLUS) were applied to the sections and cells, and incubated for 1h at 37°C. Detection of rolling circle amplification probes in cells and tissues were displayed as red punctate signals.

### Gelatin Zymography and *in situ* Zymography

Enzymatic activity of gelatinases in the odontoblastic cells and PN2 mouse mandibular molars was detected by gelatin zymography and *in situ* zymography. For gelatin zymography, cellular proteins of odontoblastic cells were subjected to electrophoresis on SDS-PAGE gels containing 1 g/L gelatin. After electrophoresis, the gels were washed in 2.5% Triton-X 100 with agitation for 1 h at room temperature (RT), followed with incubation in enzyme incubation buffer (NaCl 150 mM, CaCl_2_ 5 mM, Tris–HCl 50 mM, pH 8.0). Then the gelatin zymography gel was stained with Coomassie Brilliant Blue and de-stained. Enzyme activity was visualized as clear bands.

For *in situ* zymography, the odontoblastic cells were treated with serum-free medium for 8 h. Fresh mandibles of PN2 *WT* mouse were embedded in OCT without fixation and successive sections of 6 μm were made with freezing microtome. The experiments were performed with fluorescein conjugated gelatin as the MMP substrate (E-12055, EnzChek^TM^ Gelatinase/Collagenase Assay Kit). Gelatin mixture was made by diluting the 1.0 mg/mL gelatin stock solution with the dilution buffer (1:10), and different MMP inhibitors or DMSO were added to the mixture, respectively. The coverslips and sections were divided into four groups randomly and gelatin mixture with or without different MMP inhibitors were incubated with each coverslip or section in humidified chambers in the light-protected condition overnight. Endogenous gelatinolytic enzyme activity of the odontoblastic cells and PN2 molars was measured by detecting the fluorescence signal yielded from enzymatic digestion.

### Western Blotting Analysis

Briefly, proteins were subjected to SDS-PAGE, electrotransferred onto a PVDF membrane. Then, the membrane was blocked and incubated with each primary antibody diluted solution (DSP, 1:500; MMP9, 1:1000; FURIN, 1:5000, MYC, 1:2000) overnight, and after three washes, incubated with secondary antibody (1:4000 or 1:6000) for 1 h. Finally, Western Bright ECL (161203-21, Advansta, United States) was used to detect proteins. Band intensity was normalized using β-actin and GAPDH. The experiments were repeated more than three times.

### Immunoprecipitation

*Wild-type* odontoblastic cells were lysed with lysis buffer, and supernatants were incubated with anti-FURIN antibody at 4°C overnight. Supernatants incubated with negative IgG were served as negative control. Protein A/G magnetic beads (#710031, bimake) were used to pull-down immune complexes. Western blotting was used to analyze these eluted samples.

## Results

### DSP and Gelatinases Are Co-expressed in Odontoblasts and Odontoblastic Cells

**FIGURE 1:**
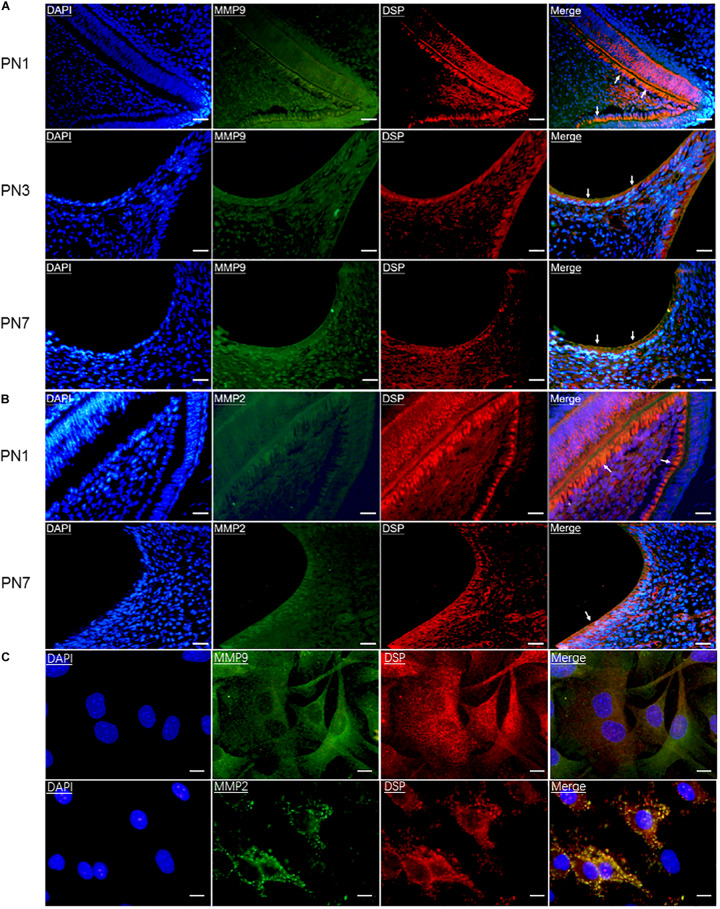
Gelatinases (MMP9 and MMP2) were co-expressed with DSP in odontoblasts and odontoblastic cells. **(A)** Double immunofluorescence showed co-immunoreactivity for MMP9 (green) and DSP (red) in the odontoblasts (arrows) of mouse molars at postnatal day 1 (PN1), PN3, and PN7. Scale bars, 100 μm. **(B)** Co-expression of DSP (red) and MMP2 (green) in the odontoblasts of mouse molars at PN1 and PN7. Scale bars, 100 μm. **(C)** Co-localization of MMP9 and DSP, MMP2 and DSP were found in the odontoblastic cells by double immunofluorescence. Scale bars, 25 μm.

To determine whether DSP was processed by gelatinases intracellularly, first, their co-expression in odontoblasts and odontoblastic cells was detected by double immunofluorescence. The results showed that DSP, MMP9, and MMP2 were all highly expressed in the pre-odontoblasts, secretory odontoblasts, and mature odontoblasts of mouse molars at PN1, 3, and 7 (Figures 1A,B). In addition, MMP9 and MMP2 were also widely expressed in dentin, dental pulp cells (Figures 1A,B), and alveolar bone (data not shown). Co-localization of DSP and gelatinases was also detected in the cytoplasm of odontoblastic cells (Figure 1C).

### DSP and Gelatinases Interact in Odontoblasts and Odontoblastic Cells

**FIGURE 2:**
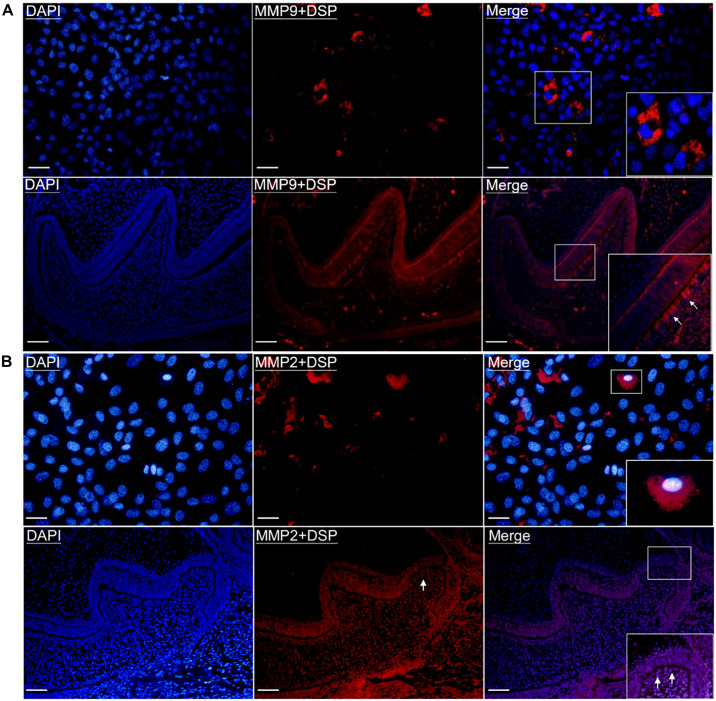
Gelatinases (MMP2 and MMP9) physically interacted with DSP in the odontoblastic cells and odontoblasts of mouse molars. **(A)** *In situ* proximity ligation assay (PLA) showed the presence of physical binding between MMP9 and DSP (red signals) in the odontoblastic cells and odontoblasts of PN2 mouse mandibular molars. Scale bars, 100 μm. **(B)** *In situ* PLA showed the interaction between DSP and MMP2 (red signals) in the odontoblastic cells and odontoblasts of PN2 mouse mandibular molars. Scale bars, 100 μm. Higher magnifications of the rectangle areas are shown. Arrows indicate the positive PLA signals in the odontoblasts.

To further determine the physical binding between DSP and MMP9/MMP2 inside odontoblasts and odontoblastic cells, *in situ* PLA was performed. As shown in Figure 2A, interaction between MMP9 and DSP was observed in the odontoblastic cells and the odontoblasts of PN2 mouse mandibular molars, as evidenced by positive PLA signals. The physical interaction between DSP and MMP2 were also present in the odontoblastic cells and the cytoplasm of PN2 mouse molar odontoblasts (Figure 2B).

### Activated Gelatinases Are Present in Odontoblasts and Odontoblastic Cells

**FIGURE 3:**
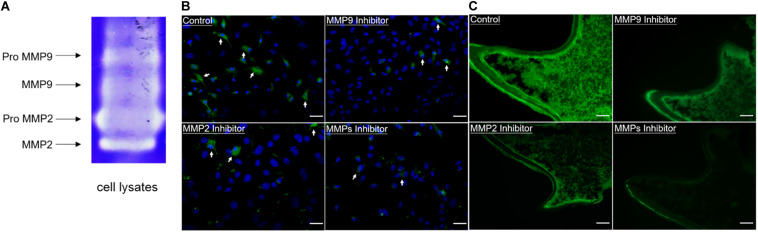
Enzymatic activity of gelatinases existed in the odontoblastic cells and odontoblasts of mouse molars. **(A)** Gelatin zymography was performed using cell lysates of the odontoblastic cells. In addition to proMMP9 and proMMP2, activated MMP9 and MMP2 were observed. **(B)** *In situ* zymography showed the presence of gelatinolytic activity (indicated by arrows) in the odontoblastic cells which was reduced by the MMP9 or MMP2 or MMP general inhibitor. Scale bars, 100 μm. **(C)** *In situ* zymography showed the presence of gelatinolytic activity in the odontoblasts, dentin matrix, and dental papilla cells, which was reduced by treatment with the MMP9 or MMP2 or MMP general inhibitor in the PN2 mouse molars. Scale bars, 100 μm.

In order to evaluate the enzymatic activity of gelatinases inside cells, gelatin zymography and *in situ* zymography were performed. Gelatin zymography showed that in addition to the latent forms of gelatinases (proMMP9 and proMMP2), activated forms of gelatinases (MMP9 and MMP2) were also present in cell lysates of *WT* odontoblastic cells (Figure 3A). As shown in Figure 3B, compared to the control group, *in situ* zymography showed that the amount of cells with fluorescence and the fluorescence intensity were decreased in the groups treated with the MMP9 inhibitor or the MMP2 inhibitor or the MMP general inhibitor. In the PN2 molars, fluorescent signals were strong in the odontoblasts and dentin without any inhibitor treatment, but obviously weakened when the tissue sections were treated with one of these inhibitors (Figure 3C).

### Full-Length DSP Is Intracellularly Cleaved by Gelatinases Into Smaller Fragments

**FIGURE 4:**
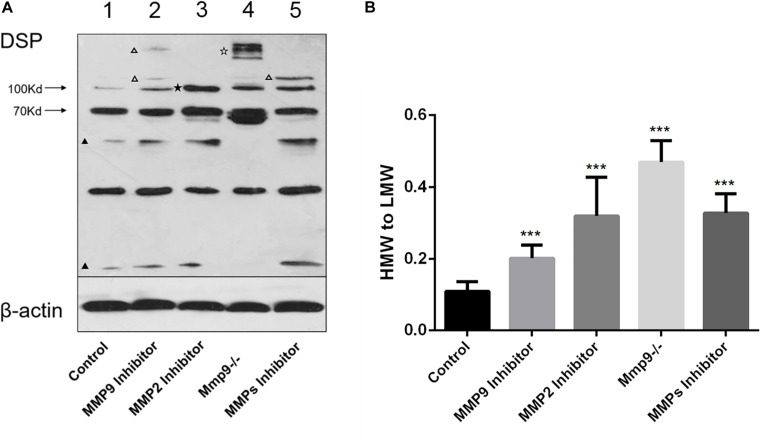
DSP was cleaved by gelatinases in the odontoblastic cells. **(A)** Proteins were obtained from *WT* odontoblastic cells treated with the MMP9 or MMP2 or MMP general inhibitor and from *Mmp9^–/–^* odontoblastic cells. Cell lysates of *WT* odontoblastic cells without treatment by any inhibitor was set as control. The protein concentration was measured and same amount of proteins in all groups were loaded. Western blotting analysis was performed using anti-DSP antibody. Different DSP protein profiles were seen in these groups. Several additional HMW DSP bands (around 250 kDa, indicated by open pentagram) appeared in the *Mmp9^–/–^* but not in the *WT* odontoblastic cell lysates, and two LMW DSP bands (lower than 70 kDa, indicated by triangles) were observed in *WT* group but not in *Mmp9^–/–^* group (lanes 1 and 4). When *WT* odontoblastic cells were treated with the MMP9 or the general MMP inhibitor, two and one additional HMW DSP bands (above 100 kDa, indicated by open triangles) appeared, respectively (lanes 2 and 5). When *WT* odontoblastic cells were treated with the MMP2 inhibitor, the density of the HMW band at 100 kDa (indicated by pentagram) significantly increased (lane 3). **(B)** The ratio of the density of HMW (100 kDa and above) to LMW (lower than 100 kDa) bands were calculated. Compared with control group, all experimental groups showed significantly higher ratios of HMW to LMW DSP. HMW, high molecular weight. LMW, low molecular weight. ***P < 0.001.

In order to prove that full-length DSP was processed by activated gelatinases intracellularly, proteins were collected from cell lysates of *WT* and *Mmp9^–/–^* odontoblastic cells, and western blotting analysis was used to measure the changes of DSP protein pattern inside the cells. The results showed intracellular expression patterns of DSP in the *WT* and *Mmp9^–/–^* odontoblastic cells were different (Figure 4A). Several HMW (high molecular weight) DSP bands (around 250 kDa, indicated by open pentagram) were observed in the *Mmp9^–/–^* cellular proteins but not in the *WT* cellular proteins, and two LMW (low molecular weight) DSP bands (lower than 70 kDa, indicated by triangles) were seen in *WT* group but not in the *Mmp9^–/–^* group (Figure 4A, lanes 1 and 4). When *WT* odontoblastic cells were treated with the MMP9 or the general MMP inhibitor, two and one additional HMW DSP bands (indicated by open triangles) appeared in the cell lysate proteins, respectively (Figure 4A, lanes 2 and 5). When *WT* odontoblastic cells were treated with the MMP2 inhibitor, the density of the HMW band at 100 kDa (indicated by pentagram) significantly increased (Figure 4A, lane 3). Compared with control group, the ratio of the HMW DSP (100 kDa and above) to the LMW fragments (lower than 100 kDa) were significantly higher in the *Mmp9^–/–^* group, and in the groups treated with the MMP9 or MMP2 or MMP general inhibitor (Figure 4B).

### FURIN Is Involved in the Activation of Gelatinases Intracellularly

**FIGURE 5:**
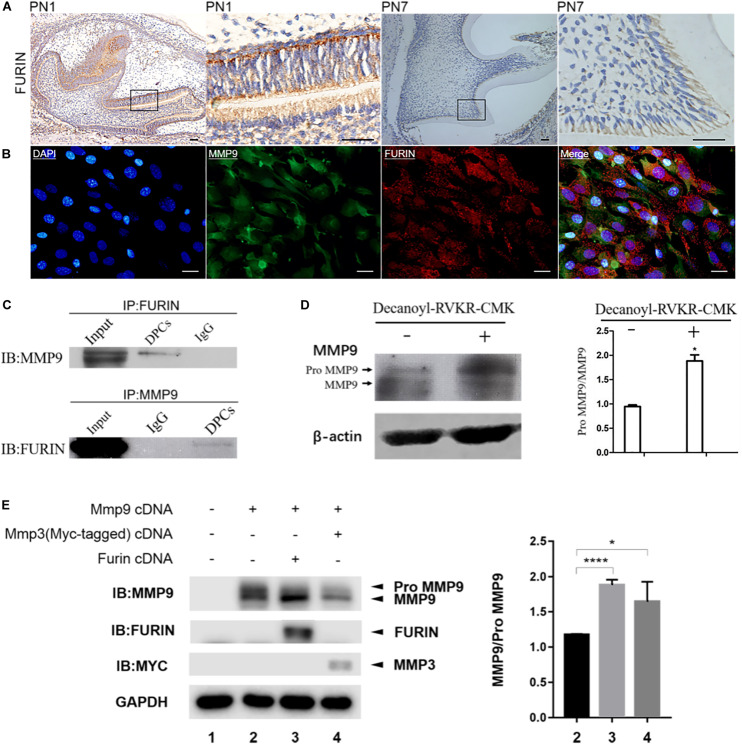
FURIN was co-expressed and interacted with MMP9 in the odontoblasts and odontoblastic cells and cleaved proMMP9 intracellularly. **(A)** Immunohistochemistry showed the strong expression of FURIN in the odontoblasts of mouse molars at PN1 and PN7. Higher magnifications of the rectangle areas are shown on the right. Scale bars, 50 μm. **(B)** Double immunofluorescence showed co-localization of FURIN and MMP9 in the odontoblastic cells. Scale bars, 100 μm. **(C)** Cell lysates of odontoblastic cells were subjected to IP with anti-FURIN antibody, and analyzed by immunblotting (IB) with anti-MMP9 antibody. IP with anti-MMP9 antibody and IB with anti-FURIN antibody were also performed. These results verified the physical interaction between FURIN and MMP9. **(D)** Protein pattern of MMP9 in the odontoblastic cells treated with (right lane) or without (left lane) Decanoyl-RVKR-CMK (a FURIN inhibitor). The ratios of proMMP9 to MMP9 were quantified and significantly higher in the Decanoyl-RVKR-CMK treated group. **(E)** HEK293E cells were transfected with *Mmp9* plasmid alone (lane 2), or together with *Furin* (lane 3), or *Mmp3* (lane 4, as positive control) plasmid. Cell lysates were collected and western blotting analysis was performed using anti-MMP9, anti-FURIN, and anti-Myc antibodies. The ratios of MMP9 to proMMP9 were calculated and significantly higher in lanes 3 and 4. *P = 0.0457 and ****P < 0.0001.

The expression of FURIN in odontoblasts and its co-localization with MMP9 in odontoblastic cells were investigated by immunohistochemistry and double immunofluorescence, respectively. FURIN was widely distributed in all cell types with an intense expression in the odontoblasts (Figure 5A). Double immunofluorescence verified the co-localization of FURIN with MMP9 in the odontoblastic cells (Figure 5B). Then, immunoprecipitation assay was performed to determine whether FURIN was able to bind with MMP9. The results showed that FURIN physically interacted with MMP9 in the odontoblastic cells (Figure 5C).

To investigate whether endogenous FURIN activated gelatinases inside odontoblastic cells, a specific FURIN inhibitor, Decanoyl-RVKR-CMK, was applied to inhibit the function of FURIN intracellularly. Compared with the control group, the ratio of latent to activated MMP9 was significantly increased in the Decanoyl-RVKR-CMK treated group (Figure 5D). *Mmp9* was overexpressed in the HEK293E cells together with *Furin* to further confirm the cleavage of MMP9 by FURIN intracellularly. MMP3 has been reported to be able to remove the prodomain of proMMP9 to activate it (Ogata et al., 1992). Therefore, *Mmp3* and *proMmp9* cDNAs were co-transfected into HEK293E cells as the positive control group. Then, cell lysates were collected, and western blotting results showed that the ratio of activated MMP9 to proMMP9 was increased when cells were transfected with *Mmp9* together with *Furin* or *Mmp3* compared with those transfected with *Mmp9* alone (Figure 5E). These results demonstrated that FURIN was able to process proMMP9 into activated form intracellularly.

**FIGURE 6:**
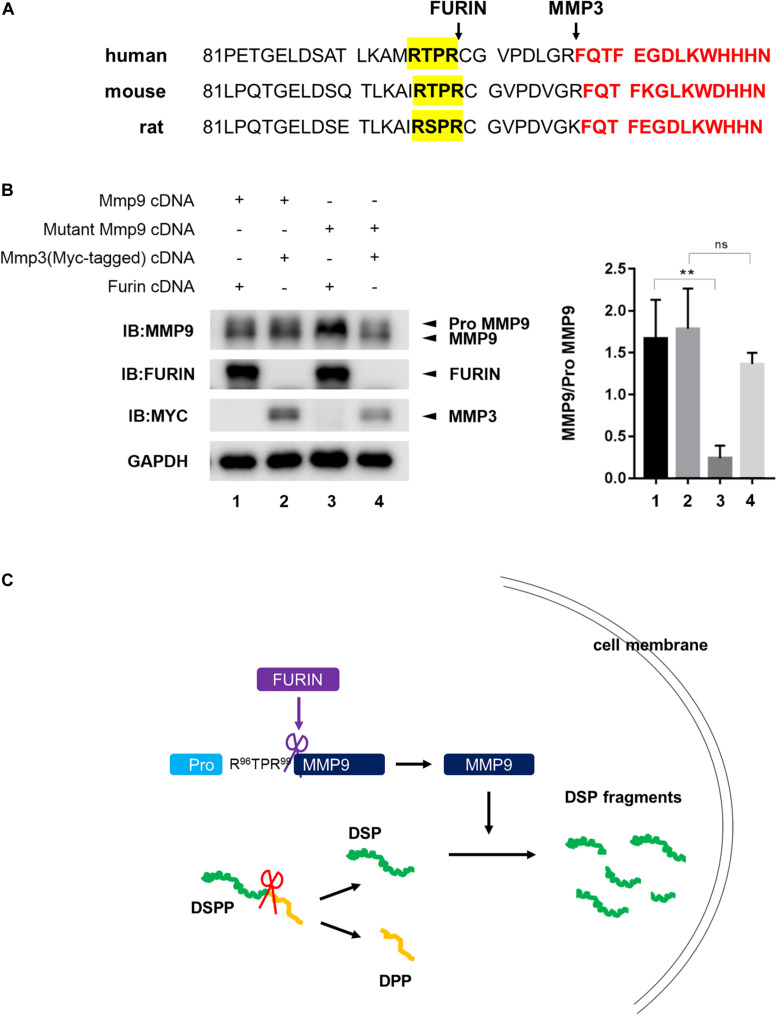
FURIN activated MMP9 through cleavage at R^96^TPR^99^. **(A)** The predicted cleavage sites of proMMP9 by FURIN in human, mouse, and rat (highlighted by yellow). The recognized cleavage sites of proMMP9 by MMP3 were also marked. Red font showed starting amino acid sequences of activated MMP9. **(B)** The proMMP9 and mutant proMMP9 with R^96^TPR^99^ substituted by A^96^AAA^99^ were separately overexpressed in HEK293E cells together with FURIN (lanes 1 and 3) or MMP3 (lanes 2 and 4). Cell lysates were examined by western blotting analysis and the ratio of MMP9 to proMMP9 was quantified. A significant lower ratio of MMP9 to proMMP9 was found in lane 3 compared with lane 1, indicating that FURIN failed to cleave the mutant proMMP9. But the ratio of MMP9 to proMMP9 did not change in lane 4 compared with lane 2, indicating that this mutation of proMMP9 did not affect its cleavage by MMP3. **P = 0.0073, ns *P* > 0.05. **(C)** Schematic model shows that MMP9 activated by FURIN cleaves DSP intracellularly.

To determine the cleavage site of proMMP9 by FURIN, the amino acid sequence of proMMP9 was analyzed. RXPR motif, a consensus substrate motif of FURIN, was found in the prodomain of proMMP9, and this motif was conserved in human (R^95^TPR^98^), mouse (R^96^TPR^99^), and rat (R^96^SPR^99^) (Figure 6A). To further confirm whether FURIN did cleave proMMP9 at the consensus sequence R^96^TPR^99^, a plasmid expressing the mutant proMMP9 with R^96^TPR^99^ substituted by A^96^AAA^99^ was constructed and overexpressed with FURIN or MMP3. The western blotting results of cell lysates showed that site-directed mutagenesis completely prevented the cleavage of the mutant proMMP9 by FURIN but did not affect its cleavage by MMP3 (Figure 6B). These results indicate that FURIN indeed directly contributes to the activation of MMP9 in the HEK293E cells, and the cleavage site is the consensus sequence R^96^TPR^99^.

## Discussion

In addition to full-length DSP, multiple DSP fragments were also detected in the cell lysates of odontoblastic MO6-G3 cells, and DSP fragments showed different distribution in the dentin matrix (Yuan et al., 2012). In our earlier study, we have also reported that DSP is a substrate of MMP9 in the tooth extracts (Yuan et al., 2017). DSPP was reported to be proteolytically processed by MMP2 during dentin mineralization and the cleavage sites of DSPP are near the DSP COOH-terminal region (Yamakoshi et al., 2006). In this study, DSP was found to be cleaved by activated MMP9 and MMP2 intracellularly and FURIN was involved in the intracellular activation of MMP9.

Because real native odontoblasts display long cell processes embedded into the dentinal tubules, the isolation of real odontoblasts will lead to damage and cell death. Therefore, induction of dental papilla cells into odontoblastic cells *in vitro* has been widely used as an excellent model for odontoblast-related experiments in many reports (Chen et al., 2015; Yang et al., 2015; Zhang et al., 2015; Iwamoto et al., 2017; Li Q. et al., 2018; Li s. et al., 2018; Zheng et al., 2020). In this study, we confirmed these cells as odontoblastic cells as described previously (Zheng et al., 2020). Co-localization of DSP and gelatinases is a prerequisite for the cleavage of DSP by gelatinases, so we first investigated the co-expression of DSP and gelatinases in odontoblastic cells and odontoblasts of mouse molars. Co-localization of DSP and gelatinases were observed in the cytoplasm of both odontoblastic cells and odontoblasts. Next, *in situ* PLA was performed to detect the possible physical interaction between DSP and gelatinases, and the results revealed that DSP interacted with gelatinases in the odontoblasts of *WT* mouse molars and in the odontoblastic cells. This is consistent with our previous finding that recombinant DSP protein was able to bind to recombinant MMP9 *in vitro*, evidenced by substrate binding assay results (Yuan et al., 2017).

Dentin sialoprotein is important for dentin formation evidenced by heterogeneous mutations in the *DSP* coding domain in humans leading to hereditary dentin defects. However, full-length DSP has little or no effect on hydroxyapatite formation or growth (Boskey et al., 2000). Our previous study found that a 36 amino acids residue of DSP domain aa^183–219^ was able to induce dental mesenchymal cell differentiation by binding to integrin β6 and activating expression of *Dspp* and *Dmp1* genes (Wan et al., 2016). In addition, DSP domain aa^363–458^ was able to regulate dental cell differentiation and mineralization by interacting with the extracellular loop 2 of occludin (Ocln) aa^194–241^ and inducing phosphorylation of Ocln (Li et al., 2017). DSP and osteopontin (OPN) are two members of the small integrin binding, N-linked glycoprotein family. OPN is processed by Thrombin and/or MMPs into COOH- and NH_2_-terminals, and this cleavage enhances the exposure of OPN active region, which can further bind to membrane receptor and activate downstream signal pathway, thereby influence cell adhesion, migration, increment, and differentiation (Agnihotri et al., 2001; Shio et al., 2010). Therefore, like the cleavage of OPN, processing of DSP by some proteases may be an activation step and important for its biological function.

Expression patterns of DSP were different between the *WT* and *Mmp9*^–/–^ teeth evidenced by western blotting of isolated proteins from mouse teeth. Several HMW DSP bands were seen in the *Mmp9*^–/–^ but not in the *WT* tooth proteins, and the ratio of the low LMW fragments to the HMW DSP was significantly lower in the *Mmp9*^–/–^ mice (Yuan et al., 2017). In this study, cellular proteins of the odontoblastic cells were collected and analyzed by western blotting. Expression pattern of DSP in the *Mmp9^–/–^* odontoblastic cells was similar to that in the teeth of *Mmp9^–/–^* mice, including the presence of HMW DSP fragments and higher ratio of HMW DSP to LMW. And when cultured odontoblastic cells were treated with MMP9 inhibitor, intracellular expression profile of DSP was also similar to that in the teeth of *Mmp9^–/–^* mice. These results indicate that the cleavage of murine full-length DSP by MMP9 into smaller fragments occurs intracellularly. DSPP is processed by MMP2 within the COOH-terminal region of the DSP, and four MMP2 cleavage products derived from DSPP have been characterized in porcine dentin (Yamakoshi et al., 2006). In the present study, the proportion of HMW to LMW DSP in the odontoblastic cells increased when MMP2 inhibitor was added to the culture medium, indicating that MMP2 also participates in cleaving murine full-length DSP into smaller subunits and the cleavage also occurs intracellularly. The processing of DSP by both MMP9 and MMP2 suggested that these enzymes may serve overlapping functions. However, in the odontoblastic cells, some new HMW protein bands (>100 KD) appeared when the cells were treated with MMP9 inhibitor while another new band (between 45 and 70 KD) emerged when MMP2 was inhibited, reminding us that these two enzymes may have distinct cleavage sites in DSP.

Several studies have demonstrated the expression of MMPs in dentin, dental epithelium, and dental mesenchyme (Palosaari et al., 2003; Yoshiba et al., 2003), but whether MMPs possess catalytic activity inside the odontoblasts have not been investigated. In this study, we revealed that activated forms of MMP9 and MMP2 were present in odontoblasts and odontoblastic cells. Palosaari et al. (2003) have detected MMP9 gelatinolytical activities (pro- and active forms) and MMP2 gelatinolytical activities (pro- and active forms) in the conditioned culture media of human odontoblasts. But in the present study, the gelatinolytical activities of MMP9 and MMP2 inside the odontoblastic cells were investigated by gelatin zymography of odontoblastic cell lysates. In addition to latent zymogens, active enzymes of gelatinases (MMP9 and MMP2) also appeared, indicating that MMP9 and MMP2 have the capacity to cleave their substrates inside the odontoblastic cells. *In situ* zymography results further confirmed the enzymatic activity of MMP9 and MMP2 inside odontoblasts *in vivo*.

In this study, FURIN was found to be co-expressed and bind with MMP9 in odontoblasts and odontoblastic cells. FURIN activates MT1-MMP intracellularly by recognizing and cleaving its RX(K/R)R (X = 0, 2, 4, 6….) motif between the prodomain and catalytic domain, and similar FURIN-mediated activation mechanisms have been detected in some other proteolytic enzymes (Pei and Weiss, 1995). The motif R^96^XPR^99^ was found between the prodomain and catalytic domain of mouse MMP9 zymogen by analyzing protein sequence. By analyzing MMP9 expression pattern in the odontoblastic cells, we found that the levels of activated MMP9 were down-regulated when the function of FURIN was inhibited by Decanoyl-RVKR-CMK, indicating that endogenous FURIN is able to activate MMP9 intracellularly. Meanwhile, overexpressed FURIN was found to be able to cleave MMP9 in the HEK293E cells. And when the consensus sequence R^96^XPR^99^ was substituted by A^96^AAA^99^, the cleavage of mutant proMMP9 by FURIN was completely abolished. Therefore, FURIN directly interacts with proMMP9 and cleaves its prodomain.

Taken together, activated MMP9 and MMP2 existed in odontoblasts, and these two enzymes cleaved full-length DSP inside odontoblasts. FURIN was involved in processing and activation of proMMP9 in the cytoplasm of the odontoblasts by cleaving its R^96^XPR^99^ motif (Figure 6C). The results of this study suggested that gelatinases may have the potential to be applied for dentin reparation after tooth injury by trauma or caries in human dentistry. Exogenous MMP9 was found to be endocytosed by mouse embryonic fibroblasts (Hahn-Dantona et al., 2001). We presumed that gelatinases in appropriate pulp-capping materials may be able to diffuse into the pulp tissue through dentinal tubules and have the potential to accelerate dentin reparation under dentin injury conditions. However, the possible adverse effects of gelatinases on the adhesive performance of the restorative materials should also be considered when developing and using the gelatinases-contained pulp–capping agents.

## Data Availability Statement

All datasets generated for this study are included in the article/supplementary material.

## Ethics Statement

The animal study was reviewed and approved by Animal Welfare Committee at the School of Stomatology, Wuhan University.

## Author Contributions

XG and YX contributed to data acquisition, analysis, drafted, and critically revised the manuscript. HZ and GYa contributed to data acquisition and analysis. SC, ZC, and GYu contributed to conception, study design, data analysis, and critically revised the manuscript. All authors contributed to the article and approved the submitted version.

## Conflict of Interest

The authors declare that the research was conducted in the absence of any commercial or financial relationships that could be construed as a potential conflict of interest.
